# Case of Emphysema of the Sub-Mucous Cellular Coat of the Stomach; with Remarks

**Published:** 1850-04

**Authors:** Francis West

**Affiliations:** Philadelphia


*Case of Emphysema of the Sub-Mucous Cellular Coat of the Stomach ; with remarks.* By Francis West, M. D., of Philadelphia.

April 20, 1840. Thomas B., white, æt. 34 years, shoemaker by trade, of temperate habits, and sound constitution, had always enjoyed good health until about a year since, when he began to complain of not feeling perfectly well, although he still continued to work at his trade. About six weeks before my first visit, according to the history which he gave me, he began to suffer from severe burning in his stomach, which steadily increased and extended throughout his abdomen, accompanied by loss of appetite, vomiting, and a sensible diminution of his urine. About a fortnight before he died, he had taken, by the advice of a Thompsonian practitioner, several powerful emetics, which operated most violently. From this time he became much worse, and then applied to a Homœopathic practitioner, who, having been only recently converted from legitimate medicine, had very wisely conjoined with his "little powders" the free use of leeches and poultices to the abdomen, with the effect of somewhat relieving him. The symptoms, however, soon became threatening again, and in view of approaching death, the patient was very willingly resigned into my hands. His condition was then as follows:—Emaciation of face considerable, of rest of body moderate; no œdema or other swelling of the integuments ; face hippocratic and expressive of severe internal uneasiness, which was constantly referred to the abdomen, pressure upon which, especially over the stomach, very much aggravated his sufferings. He said *that he felt as if there was something in his stomach which ought to come up*, and begged that he might be vomited for the purpose. His mouth was dry, with sordes about the lips and gums ; tongue also dry, hard and pointed, and abraded in spots, but without any fur upon it; voice weak, with great difficulty of swallowing, and an intense desire for cold drinks, which were freely allowed him.

He had no disposition for food, although his stomach was quite retentive; abdomen very tumid; bowels constantly open, with much pain; discharges consisting of bloody water; no cough or pain in the chest; respiration weak but natural; impulse of heart feeble; pulse about 90; skin of natural temperature; no sweating; urine not very free. A palliative treatment was advised, consisting of warm applications to the belly, with cool mucilaginous drinks, and the same kind of enemata; chicken water for nourishment. He gradually sank, as I had anticipated, and died very easily on the 23d, about 72 hours after I first saw him.

*Autopsy.*—Thirty hours after death: usual rigidity of the body; no decomposition or apparent tendency to it.

*Thorax.*—Lungs engorged with black blood; no other lesion; no effusion into the pleuræ. Heart soft and flabby, its tissue at some points being easily broken down by pressure between the fingers. Parietes thinned; valves natural; no pericardial effusion.

*Abdomen.*—The omentum was firmly adherent to the abdominal parietes at several points, and at the same time closely invested the intestines, being attached to them by false membrane, the result apparently of not very ancient inflammation. No extraordinary fetor was observed when the cavity was exposed. On laying open the stomach, attention was at once attracted to the projection into its cavity of a large, tense irregularly rounded swelling, considerably exceeding a goose egg in size, surrounded by smaller ones, similar in character, some of which were connected with the large one, whilst others were only contiguous to it; the whole resembling very much a collection of hydatids. On examination, these swellings proved to be the result of distension or inflation of the mucous membrane of the stomach, from air accumulated in its sub-mucous cellular tissue. The parietes of these inflated tumors could not be ruptured by pressure with the fingers, but on being cut, the air which escaped from them was quite inodorous. This fact was particularly noticed. The mucous membrane of the stomach was everywhere of natural firmness, and could be readily raised in strips of some length, leaving the parts beneath quite natural. No signs of inflammation were present. No part of this organ exhibited any sign of putrefactive decomposition. The intestines examined throughout, presented no appearance similar to the emphysematous condition of the stomach, neither was this latter observable in any other part of the body. The other organs were healthy.

*Remarks.*—The point of particular interest in this case is, of course, the emphysematous swellings noticed in the stomach, and in regard to them, the question at once arises, how were they produced? Was the collection of gas the result of abnormal secretion, or exhalation into the sub-mucous cellular tissue, or was it merely the effect of incipient decomposition of the part after death, or at some period nearly preceding dissolution? To the latter cause many persons may, at first view, be disposed to attribute it, and similar gaseous collections, as M. Bouillaud has done, in his article upon the subject in the *Dictionnaire de Médecine et Chirurgie Pratiques.*

From the firmness and natural condition of the rest of the stomach, and from the perfectly circumscribed character and consistence of the swellings themselves, however, as well as from the entire want of fetor in the air which escaped from them, we are more disposed to regard the emphysematous condition as the result of actual secretion, or exhalation of air into the cellular tissue during life, than as a consequence merely of putrefactive decomposition. Neither the time of year, the nature of the disease, nor the period which elapsed between death and the autopsy, seem to favor the idea of putrefaction, which, on the contrary, appears to be disproved by the entire localization of the swellings, as well as by the natural appearance of the rest of the stomach, and the entire absence everywhere else of any signs of decomposition.

May not the lesion have been connected in some way or other with the violent vomiting to which the patient had been subjected, and after which, as has been stated, he always complained of feeling something in the stomach, which, as he expressed it, "ought to come up?" The inflation of the inner membrane of the stomach to the degree noticed, might very well, as we conceive, have given rise to this peculiar sensation, and if so, we are supported in the belief that the emphysema originated *ante* and not post mortem.

Such an appearance of the stomach, we believe, has not often been noticed.

Andral, in his Pathological Anatomy, page 124, of volume 2d, under the head of "Exhalation of Gases," gives a case of the kind, as reported by Cloquet, although he evidently regards the appearance in question as one of very uncommon occurrence. Cloquet, in his history of the case, says "there was considerable emphysema of the cellular tissue uniting the different coats of the stomach, so that its parietes appeared to have been inflated, and at several points were nearly an inch in thickness."

This description accords perfectly with the one we have given, and except in Cloquet's case, we do not find any other reference in authors to emphysema of the *stomach*, although that of other organs is noticed.

Thus, Grisolle, in the first volume of his treatise on Internal Pathology, under the general head of Morbid Secretions, called by him "Pneumatoses ou Sécrétions Gazeuses," when describing "emphysema," says "that it occurs also at times in the sub-mucous cellular tissue, such as is found in the folds of the conjunctiva, and in that separating the different coats of the intestines, but more rarely in the subserous cellular tissue, especially that beneath the epiploic reflections." It is probable, he adds, that in these latter cases, the emphysema constitutes a purely cadaveric lesion.

Murat, in his article upon "Spontaneous Emphysema," in the Dict. de Médecine, or Repertoire des Sciences Médicales, says "these may possibly be referred to this sort of lesion, viz., "emphysema from exhalation," the different windy tumors, observed by Galen and Fabricius, as well as the collections of gases which appear in different parts of the body, either after, or during certain diseases, or from exposure to cold, or in some cases of poisoning, as well as from the bites of certain insects and reptiles."

